# Improving Rice Zinc Biofortification Success Rates Through Genetic and Crop Management Approaches in a Changing Environment

**DOI:** 10.3389/fpls.2016.00764

**Published:** 2016-06-06

**Authors:** Niluka Nakandalage, Marc Nicolas, Robert M. Norton, Naoki Hirotsu, Paul J. Milham, Saman Seneweera

**Affiliations:** ^1^Faculty of Veterinary and Agricultural Sciences, University of Melbourne, CreswickVIC, Australia; ^2^Faculty of Veterinary and Agricultural Sciences, University of Melbourne, ParkvilleVIC, Australia; ^3^International Plant Nutrition Institute, HorshamVIC, Australia; ^4^Faculty of Life Sciences, Toyo UniversityGunma, Japan; ^5^Hawkesbury Institute for the Environment, Western Sydney University, PenrithNSW, Australia; ^6^Centre for Crop Health, University of Southern QueenslandToowoomba, QLD, Australia

**Keywords:** biofortification, endosperm, germplasm screening, physiological mechanisms, rice, zinc deficiency

## Abstract

Though rice is the predominant source of energy and micronutrients for more than half of the world population, it does not provide enough zinc (Zn) to match human nutritional requirements. Moreover, climate change, particularly rising atmospheric carbon dioxide concentration, reduces the grain Zn concentration. Therefore, rice biofortification has been recognized as a key target to increase the grain Zn concentration to address global Zn malnutrition. Major bottlenecks for Zn biofortification in rice are identified as low Zn uptake, transport and loading into the grain; however, environmental and genetic contributions to grain Zn accumulation in rice have not been fully explored. In this review, we critically analyze the key genetic, physiological and environmental factors that determine Zn uptake, transport and utilization in rice. We also explore the genetic diversity of rice germplasm to develop new genetic tools for Zn biofortification. Lastly, we discuss the strategic use of Zn fertilizer for developing biofortified rice.

## Introduction

Dietary deficiency of zinc (Zn) is a substantial global public health and nutritional problem (Krishnaswami, 1998; Myers et al., 2014). One third of the world population is at risk due to low dietary intake of Zn (Hotz and Brown, 2004; Myers et al., 2015), including 2 billion people in Asia and 400 million in sub-Saharan Africa (Institute, 2006). Most of those at risk depend on C_3_ grains and legumes as their primary dietary source of Zn and have a high reliance on cereals, especially rice (*Oryza sativa* L.) that has a low Zn concentration with poor bioavailability compared to other cereals (Welch, 1993; Myers et al., 2014). Therefore, Zn deficiency is a chronic problem among human populations that have rice based diets (Juliano, 1993; Impa and Johnson-Beebout, 2012).

The low Zn concentration is thought to indirectly result from breeding for high yield, and for pest and disease resistance (Zhao et al., 2009). In addition, modern high yielding varieties remove large quantities of soil Zn at every harvest, lowering the residual concentration of soil Zn and contributing to lower future grain Zn concentration (Marschner, 1995; Ruel and Bouis, 1998; De Steur et al., 2014). Further, the availability of Zn for plant uptake from the soil is affected by the concentrations of macro- and micro- nutrients, the physico-chemical and biological properties of a soil (Fageria et al., 2012; Hafeez et al., 2013), as well as temperature and water availability (Weih and Karlsson, 2002; Fernando et al., 2014a). Elevated atmospheric carbon dioxide concentration (e[CO_2_]) also reduces the grain micronutrient concentration including Zn (Seneweera and Conroy, 1997a; Fernando et al., 2014b). However, in wheat, deleterious effects on grain mineral composition induced by e[CO_2_] in future might be complicated by rising temperatures coupled with increased water deficits (Fernando et al., 2014b). Any genetic and environmental interactions resulting in lower grain Zn concentration in cereals have potentially large negative implications for human health and well-being.

The aim of Zn biofortification of human food grains is to increase Zn concentration and its bioavailability in food, and this appears to be the most feasible, sustainable, and economical approach to address Zn deficiency in the human diet (Zhao and McGrath, 2009; Salunke et al., 2011; Atique-ur-Rehman et al., 2014). Biofortification could be accomplished genetically through plant breeding and agronomically through Zn fertilization. Identification of the amount of genetic variability for Zn concentration in the germplasm is the initial step, then improving rice Zn concentration (Anuradha et al., 2012). Further, a sound understanding of Zn uptake, root to shoot translocation, distribution and grain loading is essential to achieve the biofortification target.

Limited progress has been made to increase the Zn concentration in rice grain through biofortification despite a large effort, an outcome that may be a consequence of incomplete understanding of the physiological and molecular mechanisms of Zn uptake and utilization, and its environmental interactions (Anderson et al., 2001; Jiang et al., 2007; Shehu and Jamala, 2010; Gao et al., 2011; Ishimaru et al., 2011). In general, internal Zn levels of plants are controlled by a number of mechanisms in which Zn transporters play an important role. However, there is limited information on the long distance Zn transport in the plants. On the other hand, transporters of divalent metal cations also play an important role in Zn uptake, but those transporters show broad substrate specificity, so that deficiency in calcium (Ca), iron (Fe), copper (Cu), manganese (Mn), or magnesium (Mg) may result in enhanced uptake of Zn, which could lead to higher grain Zn concentration (Alloway, 2008; Hafeez et al., 2013). This review will focus on the importance of rice as a source of Zn for mankind, the major focus being on the key limitations to Zn biofortification, particularly uptake, transport and utilization. It will also explore the genetic and environmental impact on Zn biofortification using rice as a model plant for research in monocots or cereals.

## Zinc and Human Health on a Global Perspective

Zinc has multiple roles in the human body including the efficient functioning of cellular metabolic activities and stimulation of the immune system. Zinc is also present in nearly 300 enzymes in the human body (Anderson et al., 2001; Barnett et al., 2010), is important for bone mineralization, the growth of body tissues and the fetus, sperm production and fertility, smell, vision, taste and appetite, healthy growth of skin, hair and nails, as well as blood clotting and wound healing, functioning of the immune system and thyroid, cell division, protein and DNA synthesis. Daily intake of Zn is important as the mammalian body has limited Zn stores and the daily requirement is influenced by gender and physiological stage (Food and Board, 2001).

Zinc deficiency is recognized as one of the major nutrient disorders in humans and its effects are more profound in children (Boonchuay et al., 2013). Zinc deficiency is responsible for the development of a large number of illnesses and diseases including stunting of growth, compromised immune system function (Prasad, 2009; Barnett et al., 2010), cancer (Hotz and Brown, 2004), susceptibility to infectious diseases, iron deficiency anemia, and poor birth outcomes in pregnant women (Prasad, 2009; Graham et al., 2012), hair and memory loss, skin problems, weakening of body muscles, infertility in men, and pneumonia in children (Stein et al., 2005; Das and Green, 2013). Impaired Zn homeostasis is associated with several diseases, including diabetes mellitus (Jansen et al., 2009; Wijesekara et al., 2009; Foster and Samman, 2010) and zincuria which is one of the symptoms of diabetes (McNair et al., 1981; Cunningham et al., 1994; Wijesekara et al., 2009). Zinc supplementation amends glycemia in both type 1 and type 2 diabetes (Chen et al., 2000; Anderson et al., 2001; Simon and Taylor, 2001). Zinc can be supplemented thorough dietary sources such as seafood, meat, green leafy vegetables and grains. However, maintaining a sufficient Zn concentration in rice grain is important for more than half of the world population for whom rice is the staple diet.

## Rice as Source of Zn

Rice is one of the most important global staple food crops with a very long history of cultivation. On average, the grain comprises 80% starch, 7.5% protein, 0.5% ash, and 12% water. The average adult in China and India ingests about 300 g of raw rice per day (Popkin et al., 1993; Krishnaswami, 1998) and annual consumption is 62–190 kg year^-1^ (Lu et al., 2008). The daily Zn requirement is 15 mg for both adults and children that are 4 and older, but this cannot be achieved through a typical rice-based vegetarian diet (Lu et al., 2008). Though rice is the predominant source of energy, protein and micronutrients for more than 50% of the world population, it does not provide enough essential mineral nutrients to match human requirements.

The amount of mineral nutrients in rice grain is a key determinant of its nutritive value (Anuradha et al., 2012). Brown rice comprises 90% endosperm, 6–7% bran and 2–3% embryo on average by weight (Chen et al., 1998). Bran is the major repository for lipids, proteins, vitamins, minerals, and dietary fiber compared to the endosperm (Sun et al., 2010; Hoekenga, 2014; Shahzad et al., 2014). Recent X-ray micro-fluorescence investigations demonstrated that the concentrations of Zn, Fe, and potassium (K) decrease in the order: bran > hulls > whole grain > brown rice and polished rice (Johnson, 2013; Lu et al., 2013). Zinc is distributed throughout the endosperm (polished rice; Takahashi et al., 2009; Johnson, 2013), which because of its relatively large mass accounts for ∼75% of grain Zn (Wang K.M. et al., 2011). Since the Zn concentration in bran is ∼3 times greater than that in the hulls and endosperm (Trumbo et al., 2001; Lu et al., 2013), and dehulling and polishing of rice removes bran from the grain, polishing rice depletes the very element that is deficient in the diets of many of its consumers. Polished rice grains supply only one fifth of daily Zn requirements (Prom-u-thai et al., 2010; Sharma et al., 2013). Therefore, it is important to increase the Zn concentration in rice endosperm, and this can only be achieved by understanding of the genetics of Zn uptake, remobilization, transport in the plant and environmental interactions on these processes.

## Key Determinents of Grain Zinc Concentration

Rice grain Zn concentration is affected by a large number of plant and environmental factors (Welch and Graham, 2002). Plant factors affect the uptake, transport and remobilization of Zn to developing grains (Wissuwa et al., 2008). The uptake and storage of nutrients are influenced by tissue demand, plant age and the root system, but all depend on the genetic makeup (Fageria, 2013). Environmental variables that influence the Zn concentration of rice grains include soil Zn status, temperature and atmospheric [CO_2_] (Seneweera et al., 1996; Welch and Graham, 2002; Fernando et al., 2012, 2014b). There is limited understanding of how these plant and environmental factors influence and interact to affect Zn uptake, transport and loading into the grain. Thus two major questions arise for the development of a rice biofortification program, namely the extent to which the major determinants of grain Zn concentration are: (i) physiological and genetic mechanisms, or (ii) available soil Zn and its management. These propositions are dealt with in detail below.

### Zn Uptake and Translocation in Rice

Lowland rice is grown under continuously submerged conditions where low availability of Zn has been widely reported (Fageria, 2013; Meng et al., 2014). Zinc in the soil solution is transported toward the roots by mass flow and the amount intercepted is increased by diffusion and root extension (McGrath and Lobell, 2013; Yoneyama et al., 2015). Rice roots absorb Zn (Dobermann and Fairhurst, 2000; Yoneyama et al., 2015) as the divalent cation, Zn^2+^, or as its complexes with organic ligands, via different transporter systems (Suzuki et al., 2008; Yoneyama et al., 2015). Most Zn is taken up by active transport and the energy demand is largely supported through the light reactions of photosynthesis (Behrenfeld et al., 2004; Yoneyama et al., 2015). The ZIP (Zinc-regulated transporters, Iron-regulated transporter-like Protein) family of transporter genes *OsZIP1* and *OsZIP3* are involved in Zn uptake in rice (Bashir et al., 2012; Humayan Kabir et al., 2014). The molecular aspects of this response are now partly elucidated, but genetic and environmental impacts on ZIP family genes have not been explored. Moreover, in the *Arabidopsis thaliana* genome, a large number of cation transporters potentially involved in metal ion homeostasis have been identified in Zn transport (Mäser et al., 2001) and their role in Zn uptake in rice is yet to be explored.

**FIGURE 1:**
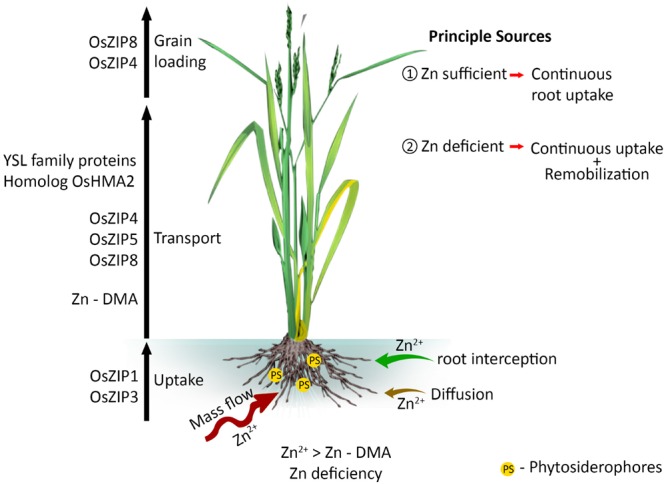
**Mass flow of Zn - uptake and transport to loading into the rice grain.** Different Zn transporters are involved in long distance transport and this flow differently regulated by Zn availability in soil and status in plants.

Zinc taken up by roots is transported to vascular tissues through the epidermis, cortex and endodermis (Marschner, 1995; Yoneyama et al., 2015), and both symplastic and apoplastic pathways play an important role (White et al., 2002; Sadeghzadeh, 2013). During symplast to symplast movement, Zn enters the apoplast before it is acquired by the new symplast (Olsen and Palmgren, 2014). The fundamental role of membrane bound proteins in Zn translocation across tonoplast, chloroplast, and plasma membranes is well recognized (Olsen and Palmgren, 2014). The negative plasma membrane potential energetically favors the import of Zn over its export (Olsen and Palmgren, 2014). Zinc occurs in the xylem complexed to small proteins (soluble form) or phytate, and its movement is driven by the transpiration stream (Sadeghzadeh, 2013). Zinc in the phloem is coupled with nicotianamine (NA), which is the predominant ligand in rice phloem sap (Nishiyama et al., 2012). Not all the Zn taken up by roots is immediately transported to the shoots, part being stored at the basal node, which may play a major role in determining Zn distribution (Sasaki et al., 2015). The ZIP family transporter genes of *OsZIP4, OsZIP5*, and *OsZIP8* are involved in root to shoot Zn transport (**Figure 1**). OsZIP3 is also involved for the unloading Zn from the xylem of enlarged vascular bundle and regulates the distribution of Zn to the developing tissues (Sasaki et al., 2015). Despite the contribution of ZIP in Zn homeostasis, homolog *OsHMA2* (P-type heavy metal ATPase) is also engaged in root-to-shoot transport of Zn in rice (Satoh-Nagasawa et al., 2012; Takahashi et al., 2012). *OsHMA2* is also involved in preferential distribution of Zn to the developing tissues by co-operating with OsZIP3 (Sasaki et al., 2015).

Zinc allocation between plant organs plays an important role in determining grain Zn concentration. Zinc allocation among different plant parts appears to be largely influenced by physiological growth stages and the nutrient status of the rice plant (Seneweera, 2011; Impa et al., 2013a; McGrath and Lobell, 2013; Shivay et al., 2015; Yoneyama et al., 2015). Depending on tissue demand, Zn is firstly allocated to leaves during vegetative growth and to grains during grain filling, i.e., the highly metabolically active sinks, and subsequently to the stem and sheath (Jiang et al., 2008). At the later stage of development, Zn partitioning between the grain, leaf blade, and shoots also varied (Seneweera, 2011). In general, to achieve maximum growth potential, the critical minimum Zn concentration is 20–25 mg kg^-1^in the dry flag leaf blade (Reuter and Robinson, 1997). At lower Zn concentrations, grain yield decreases sharply, thus it is important to maintain at least the critical internal Zn concentration. Therefore, assessment of critical nutrient requirements of Zn efficient and inefficient cultivars will provide new insights into Zn homeostasis mechanisms.

The distribution of Zn within the rice plant is associated with several step-wise processes that involve both the xylem and phloem (Impa and Johnson-Beebout, 2012; Impa et al., 2013a,b). It has been suggested that Zn translocation from roots to shoots, and older to new leaves, is associated with Zn use efficiency (Graham and Rengel, 1993), and thus grain Zn concentration may be determined by transport from the roots, stems and leaves (Jiang et al., 2007; Stomph et al., 2009). Phloem mobility of Zn from leaves to rice grain is considered to vary depending on genotype (Impa et al., 2013a). However, there has been little exploration of the complex genetic traits associated with Zn uptake and transport, particularly the role of remobilization in Zn loading into the grain.

### Zn Remobilization and Loading into the Grain

Grain Zn is derived either through root uptake (subject to soil Zn availability and root activity during the grain-filling phase) or by internal remobilization, where the rate of leaf senescence plays an important role (Arnold et al., 2010; Impa and Johnson-Beebout, 2012). These factors may explain part of the genetic variation in grain Zn concentration (Sperotto, 2013), which can be large (Meng et al., 2014), but they are not fully investigated. Under ample soil Zn conditions, direct root uptake contributes the major portion of grain Zn for most of rice genotypes (Jiang et al., 2008; Sperotto, 2013). When Zn uptake is dominant, Zn concentrations in leaves, sheaths and roots remain unchanged or continue to increase during senescence (Impa et al., 2013b). Unlike wheat, there is no xylem discontinuity in rice (Zee, 1971; Krishnan and Dayanandan, 2003), and Zn is transported directly from the stem into the rachis and vascular bundle of grains through the xylem (Jiang et al., 2008). Within the rice grain, the apoplastic pathway plays an important role in Zn transport from the nucellar epidermis and aleurone cells to the endosperm (Jiang et al., 2008).

Large amounts of grain micronutrients may remain in the outer aleurone layers of the grain (Wang K.M. et al., 2011; Myers et al., 2014) and the reasons why this Zn is not loaded into the endosperm are not well understood. If the major target is biofortification, the mechanism(s) that determine the allocation of Zn between the aleurone layer and inner endosperm need to be resolved. There is evidence that genotypic variation in Zn partitioning between the endosperm and the aleurone layers may be due partly to differences in Zn loading into the inner endosperm (Yang et al., 1998; Impa et al., 2013b). It has been suggested that Zn readily transports from aleurone to the inner part of the endosperm, particularly during early grain growth, indicating that there is no particular restriction at that time (Wang Y. et al., 2011). However, later during grain filling, Zn transport is inhibited, particularly once starch is laid down (Wang K.M. et al., 2011). By this time large amounts of phytic acid (PA) have accumulated in the outer aleurone layer. The role of PA in Zn transfer to the endosperm is not well understood.

When the soil Zn is deficient and its uptake is low, Zn remobilized from the leaves, stems, and roots is the main source of grain Zn (Impa et al., 2013a; Sperotto, 2013). Transporter genes such as *OsZIP4* and *OsZIP8* play key roles in grain Zn loading (**Figure 1**). These genes are likely to be influenced by factors such as temperature, pH and other micro-nutrients (Grotz et al., 1998; Ning et al., 2015; Yoneyama et al., 2015). For example, in *Arabidopsis*, ZIP transporters only increase the rate of Zn uptake in acidic conditions (Ramesh et al., 2003). However, there is limited understanding on how soil pH influences the expression of ZIP family genes in rice.

### Phytosiderophores in Zn Homeostasis

Phytosiderophores (PS) are non-protein amino acids produced as root exudates by graminaceous species as a response to Fe and Zn deficiency (Zhang et al., 1991; Kochian, 1993; Marschner, 2012; Humayan Kabir et al., 2014; Yoneyama et al., 2015). These complex organic ligands have been identified for their ability to improve the uptake of metals from the rhizosphere and further, facilitate internal metal transport (Impa and Johnson-Beebout, 2012). One group of PS produced by graminaceous plants is the mugineic acid (MA) family PS (2-deoxymugineic acid, 3-hydroxymugineic acid, and avenic acid). MA family PS exhibit high binding affinity with divalent metal cations including Zn^2+^ (Impa and Johnson-Beebout, 2012). ZnPS (Zn phytosiderophose complex) have greater ability to complex with Zn and improve Zn mobility in the rhizosphere to the root apoplast (Zhang et al., 1991; Yoneyama et al., 2015). Among them, DMA (2’-deoxymugineic acid) is the dominant PS released from the roots of rice plants (Bashir et al., 2006). It has been reported that exudation of DMA was greater in rice lines tolerant of Zn deficiency than in susceptible lines (Fan et al., 2001). Further, there was a strong concentration gradient of PS away from the root surface, with an average of 1 mM within the soil first 0.25 mm during the maximum exudation rates in Zn deficiency tolerant lines (Römheld, 1991). Zn-DMA plays a key role in long distance transport of Zn under Zn deficient condition in rice (Suzuki et al., 2008) and also identified for its ability to improve the availability of metals in the rhizosphere (Impa and Johnson-Beebout, 2012).

It has been reported that PS secretion is diurnally regulated, reaching a peak 2–3 h after sunrise (Takagi et al., 1984; Nagasaka et al., 2009). Mugineic acid family PS production has now been well documented in wheat (Cakmak et al., 1994), and barley (Suzuki et al., 2006; Nozoye et al., 2011) in response to Zn deficiency. Recent isotope fractionation studies and mathematical modeling support the concept that PS release is the major mechanism explaining the differences in Zn uptake by rice genotypes with varying uptake efficiency (Arnold et al., 2010). However, natural genetic variation in PS release kinetics and Zn absorption by rice roots are not well understood, and alterations in the quantity of and the efficiency of PS release exhibits both intra- and inter-specific variation. Rate of DMA release by Zn efficient genotypes is very much faster when compared to their counterparts; however, release does not appear to be stimulated under extreme Zn deficiency in either efficient or inefficient genotypes (Widodo et al., 2010). It is likely that the improvement of Zn uptake efficiency seen in plants growth under low Zn supply is associated with DMA production.

Phytosiderophores could also improve Zn availability from ions bound to/in iron plaque and thus increase Zn uptake (Impa and Johnson-Beebout, 2012). Enhanced rhizosphere Zn bioavailability due to the presence of PS could be an important mechanism for reducing Zn deficiency in lowland rice (Gao et al., 2011). Very few studies have attempted to elucidate the mechanism of PS production and Zn acquisition, and the role of PS on grain Zn loading has not received sufficient attention. Thus future studies should focus on improving the understanding of Zn loading into the grains under varying levels of soil Zn status.

### Plant Available Zn in Soil

Genetic factors aside, plant available soil Zn is recognized as one of the key factors contributing to grain Zn concentration, and this can be further increased by supplementation with Zn fertilizers (Ishimaru et al., 2011). About 30% of the cultivable soils (Hacisalihoglu and Kochian, 2003; Alloway, 2008; Anjos et al., 2012) and about 50% of the cereal growing soils in the world are low in Zn (Graham and Welch, 1996; Cakmak et al., 1999), which has serious consequences on crop growth, and consequently on human and animal health.

It is widely known that soil Zn solubility and availability are restricted by numerous factors including high CaCO_3_, high pH, high soil P concentration, high clay, low soil organic matter, and high concentrations of Fe and aluminum oxides (Weerasuriya et al., 1993; Alloway, 2008; Cakmak, 2008). The diagnosis of Zn deficiency depends on plant tissue tests because soil tests for bioavailable Zn are generally unreliable (Rayment and Lyons, 2010). Other macro- and micro- nutrients, temperature and the biological properties of the soil may also be involved in Zn bioavailability (Fageria et al., 2012; Hafeez et al., 2013; Atique-ur-Rehman et al., 2014). Inherently low Zn status in soil is aggravated by submerged conditions (Johnson-Beebout et al., 2009); consequently lowland rice is more susceptible to Zn deficiency than other cereals. During flooding or inundation (anaerobic conditions), plant available Zn decreases as a result of the formation of insoluble compounds like Zn(OH)_2_ and ZnS (Alloway, 2008). Aerobic conditions reverse these effects and the oxidation of soil organic matter can release Zn^2+^, but high levels of organic matter and clay particles could lead to Zn deficiency because Zn^2+^ binds with humic substances (Katyal and Randhawa, 1983; Hafeez et al., 2013). However, there is limited understanding of the interactive effect of soil organic matter and clay on Zn availability. Further, uptake and translocation of Zn in rice are significantly and positively correlated with cadmium (Cd) uptake (Liu et al., 2003). Higher dietary Cd is a concern because many populations for which rice is the staple carbohydrate are already over-exposed to Cd.

### Rising [CO_2_] and Grain Zn

The atmospheric concentration of CO_2_ ([CO_2_]) is rising rapidly and the current level of 400 μmol mol^-1^ (NOAA, 2013) is predicted to reach 550 μmol mol^-1^ by 2050 (Carter et al., 2007) and 970 μmol mol^-1^by the end of the 21st Century (IPCC, 2007). Effects of elevated [CO_2_] (e[CO_2_]) on the climate and on food production have become a major concern for global food and nutrient security. Importantly, e[CO_2_] is likely to have a profoundly affect on plant growth, yield, and grain quality (Seneweera, 2011; Seneweera and Norton, 2011; Fernando et al., 2014b, 2015; Myers et al., 2014; Thilakarathne et al., 2014) including rice (Seneweera, 2011; Seneweera and Norton, 2011; Myers et al., 2014). Without nutrient and water limitations, e[CO_2_] increases yield by enhancing photosynthesis and reducing crop water use (Hasegawa et al., 2013). In addition, substantial reductions in grain quality of a number of species including rice and wheat have been reported under e[CO_2_] (Seneweera et al., 1996; Högy et al., 2009; Kant et al., 2012) suggesting that e[CO_2_] alters the balance between carbon metabolism, and nutrient uptake and utilization. In wheat, decreased grain protein, and changes in protein quality, starch properties and in micronutrient densities become more prominent under this scenario (Högy and Fangmeier, 2008; Fernando et al., 2012). Overall concentrations of most of the macro- and all micro- nutrients declines as a consequence of e[CO_2_] (Myers et al., 2014). Despite the well documented physiological effects of e[CO_2_] on growth and biomass of rice, information is scant regarding the mechanisms that mediate the effects of e[CO_2_] on mineral concentrations, especially of Zn.

A recent meta-analysis showed that e[CO_2_] reduces the concentration of nitrogen (N), P, Ca, Mg, Zn, and Cu in the grain of most important cereal crops including rice (Myers et al., 2014). Others have reported 2–20% declines in concentrations of Mg, Zn, and Fe in crop species including rice, wheat and barley under e[CO_2_] (Fangmeier et al., 1999; Zuther et al., 2004; Myers et al., 2014). In a free air [CO_2_] enrichment (FACE) study using wheat under low rainfall, there was an overall reduction in the concentration of grain Zn (22%) and Fe (10%), and there was a strong positive correlation between protein and these three elements (Fernando et al., 2014b). In another FACE experiment with wheat, grain Zn and Fe declined by 13–23 and 18.3%, respectively (Högy and Fangmeier, 2008; Erbs et al., 2010). Similarly, 25% reductions in grain Fe and Zn concentrations of two wheat cultivars were found under open-top chambers at 718 μmol mol^-1^ (Högy and Fangmeier, 2008). However, it is largely unknown whether this nutrient depletion in wheat is associated with suppression of nutrient uptake, transport into the grain or a reduction in grain loading. Explanations include: biomass dilution (Poorter et al., 1997), reduction in transpiration, altered root architecture (McDonald et al., 2002) and changes in micronutrient requirements (McGrath and Lobell, 2013). Micronutrient dilution due to higher soluble carbohydrate and starch at e[CO_2_] do not fully explain lower Zn concentrations in wheat grain, because decreases in Zn concentration were not always associated with yield increases (Fernando et al., 2014b). Altered translocation of minerals to grain may also play a major role and could explain why the concentrations of some macro-elements, such as K and P, increased at e[CO_2_]. However, there is limited understanding of whether physiological demand for Zn was reduced or another mechanism played a major role in Zn partitioning into the wheat grain at e[CO_2_].

Elevated [CO_2_] increases soluble sugars and starch accumulation in leaves, and the supply of metabolically active sugars influences metabolic and cellular functions by changing gene expression (Van Oosten and Besford, 1996; Gupta et al., 2005). This could work in two different ways. Either the depletion of sugars leads to activation of gene expression, or when output exceeds the capacity of the plant to metabolize or export sugars, the resulting increase in sugar concentrations in the leaf triggers repression of genes (Van Oosten and Besford, 1996). These two distinct effects, however, are probably the result of a single regulatory mechanism, as enhanced expression following sugar depletion seems to be largely the result of de-repression of sugar controls on transcription (Koch et al., 1996). Thus, we speculate that sugar accumulation under e[CO_2_] may suppress the expression of ZIP genes and cause the observed decline in Zn uptake. Currently, our laboratory is investigating how increased sugar aﬄux to roots at e[CO_2_] affects nutrient uptake and transport as well as gene expression related to Zn metabolism.

### Fertilizers as a Grain Zn Determinant

There is increasing evidence that improved growth, yield, and grain Zn concentration could be achieved through Zn fertilization of many crops, including rice (Shehu and Jamala, 2010; Fageria et al., 2011). Thus, it is important to ensure that there is adequate Zn supply, either by soil Zn fertilization or foliar Zn application at critical growth stages such as heading and early grain-filling (Boonchuay et al., 2013; Mabesa et al., 2013).

Nitrogen and P applications could also considerably influence grain Zn concentration of rice because N application during grain filling promotes Zn uptake and remobilization (Erenoglu et al., 2011; Kutman et al., 2012; Khan et al., 2015). It has been suggested that synchronizing both Zn and N fertilization might achieve better results than sole application by avoiding the dilution effect (Alloway, 2008). Although, high rates of P application may improve shoot growth and grain yield of rice (Fageria, 2013), it may slow Zn uptake by increasing Zn adsorption to soil particles and reducing Zn absorption (Alloway, 2008). Most of the Zn use in the field is zinc sulfate fertilizer, which is the most common Zn fertilizer used on rice, but which has also been shown to be one of the least effective (Shivay et al., 2008). It would be useful to investigate how other types of Zn fertilizers improve the Zn bioavailability for the plant. Further, development of improved formulations and delivery methods for Zn application to rice is urgently needed.

On other hand, dissolved humic substances can complex Zn in soil solution, which can either make Zn less available to plants compared with sorption to cation exchange sites (common in aerobic soils) or more available to plants compared with precipitation of Zn as sulfides or carbonates (common in anaerobic soils; Mandal and Mandal, 1986). Higher OM also tends to drive redox potential down faster upon flooding because it provides an additional C source for microbial activity, which can cause the low-redox potential precipitation reactions to happen sooner and make Zn less available to rice plants (Yoshida and Tanaka, 1969). These findings suggest that using Zn fertilizers requires a good understanding of soil conditions, but there is little information on the interaction of genotypes and fertilizer use. Recently, it has been reported that nanoparticles of titanium dioxide and ZnO boost nutrient concentration and growth of tomato plants (Raliya et al., 2015). The mechanisms and physiological impact of nanoparticle uptake and translocation should be unraveled. Irrespective of the genotypes used and any differences in Zn efficiency, removal of Zn in grain depletes soil Zn, which must be replaced.

As described in section “Rising [CO_2_] and Grain Zn,” e [CO_2_] reduces both Zn and Fe concentrations in rice grains relative to other micronutrients, and that the negative effect on Zn may be greater if P is in higher supply (Seneweera and Conroy, 1997b). These findings emphasize the importance of maintaining soil fertility to improve, or at least to maintain, existing levels of grain micronutrients, especially Zn and Fe, under e[CO_2_].

### Anti-Nutrients and Zn Bioavailability

Bioavailability of nutrients in rice is largely determined by the concentration of chelating molecules in the grain such as PA (Sperotto, 2013), which is the most abundant anti-nutrient in cereal grains. In cereals more than 80% of P is invested in PA which binds metallic ions such as Fe, Zn, K, Mg, and Mn and thus reduces their bio-availability (Cakmak, 2008). For example, doubling the concentration of PA from 400–500 to 1000 μmol halved Zn absorption from >10 to >5% in feeding studies with rats (Sandström and Lönnerdal, 1989; Ruel and Bouis, 1998). These findings suggest that the bioavailability of nutrients can be improved by decreasing the PA level in the grain.

Although PA suppresses Zn bioavailability, increased PA levels in grain have positive effects on plant growth and development (Yoneyama et al., 2015). During seed germination, PA is hydrolysed to release phosphate, inositol and micronutrients to support the emerging seedling. Phytic acid and its derivatives are also implicated in RNA export, DNA repair, signaling, endocytosis and cell vesicular trafficking. On the other hand, PA consumption provides protection against a variety of cancers mediated through interruption of cellular signal transduction and cell cycle inhibition activity (El-Sherbiny et al., 2001). It has therapeutic use against diabetes mellitus, atherosclerosis and coronary heart disease and reduces kidney stone formation, HIV-1 and heavy metal toxicity (Lee et al., 2006).

In rice grain, PA is localized in the aleurone layer, and Zn in the endosperm is assumed not to be complexed with PA (Iwai et al., 2012). Understanding of the physiological and genetic regulation of tissue localization for both PA and Zn is indispensable to achieve Zn biofortification without losing the physiological effectiveness of PA.

## Ways To Overcome Global Dietary Zn Deficiency

While it may seem more efficient to directly supplement dietary Zn, this solution is unlikely to be adopted because of cost. Because Zn malnutrition occurs predominantly where poverty is high and accessibility is difficult, those at most risk of deficiency are also those least able to purchase these dietary supplements. A more appropriate strategy is seeking interventions that can raise the concentration of Zn in dietary staples.

**FIGURE 2:**
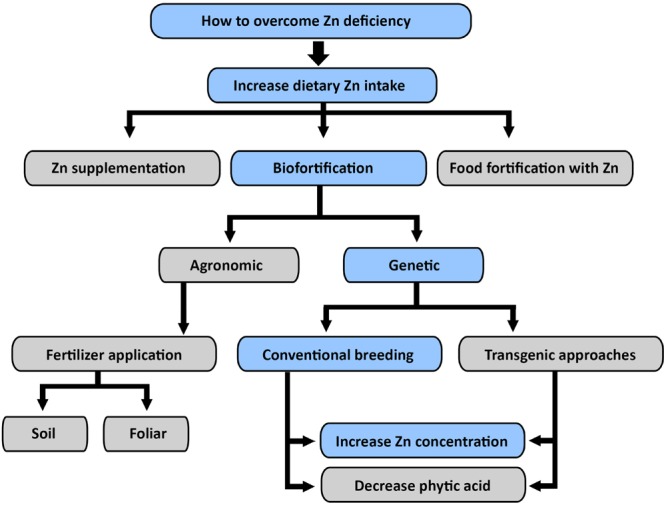
**Different approaches to overcome dietary Zn deficiency: genetic biofortification through conventional breeding appeared to be sustainable and economically viable approach to overcome dietary Zn deficiency in global population.** Blue color is used to indicate the authors’ preferred priorities for further biofortification work.

To achieve Zn biofortified grain, greater understanding of the genetic and environmental interactions in controlling Zn homeostasis in rice is urgently needed (Welch and Graham, 2002). The global Zn nutrition goal will require the deployment of a variety of strategies including: biofortification by genetic engineering or conventional breeding after screening and genetic analysis of under-utilized rice cultivars, alongside nutrition education and promotion (Kennedy et al., 2002). For example, increased Fe concentration of rice endosperm was achieved through over expression of nicotianamine synthase genes (NAS), or ferritin in conjunction with NAS genes. The single-gene approaches increase Fe concentration two-fold and the multi-gene approaches sixfold. Further, it suggested that *OsNAS* genes, particularly *OsNAS2* have great potential for Fe and Zn biofortification of rice (Johnson et al., 2011). There is evidence that over expression of *A. thaliana* Zn transporter in barley (*Hordeum vulgare* L.) doubled the grain Zn concentration (Ramesh et al., 2004), but there are issues with acceptance of genetically modified rice among consumers (Pintasen et al., 2007) because ecological considerations of moving barley genes into the *Oryza* gene pool (Lu and Snow, 2005). Unlike transgenic approaches for biofortification of vitamin A and Fe, it appears that conventional breeding approaches are much more practical in breeding Zn enriched rice grain (**Figure 2**).

## Conventional Breeding as an Effective Tool for Zn Biofortification

Production of high yielding rice varieties has been the major focus of rice breeding programs and selection of rice with high grain micronutrient concentrations has largely been ignored as a breeding objective (Graham and Welch, 1996). However, greater emphasis is now being given to nutritional aspects since micronutrient deficiency, especially of Zn, has become a well-recognized globally (Welch, 1993; Seneweera et al., 1996; Kennedy et al., 2002; Seneweera, 2011; Kant et al., 2012; Myers et al., 2014). The HarvestPlus program is an international initiative that aims to address human micronutrient malnutrition through improving the micronutrient concentration of staple foods. This program has targeted grain Zn levels of brown and polished rice, respectively, of 30 and 28 mg kg^-1^. Achieving these targets will require strategic use of Zn fertilizers, as many rice fields have low available Zn levels (Johnson-Beebout et al., 2009). So, a combination of genetic and agronomic strategies is required to raise grain Zn concentration. The genetic approaches can be advanced through germplasm screening of old landraces, traditional varieties and wild species to create novel genetic tools to increase Zn concentration in rice grain.

Germplasm screening is the initial step for a breeding program to raise grain Zn concentration and to achieve breeding objectives there should be a wide genetic variation in grain Zn concentration. In addition, substantial genetic variation of Zn concentration in brown rice (13.5–58.4 mg kg^-1^) has been reported for a large collection of rice germplasm at the International Rice Research Institute (IRRI), with an average of 25.4 mg Zn kg^-1^ (Welch and Graham, 2002; Boonchuay et al., 2013). The world’s first Zn enriched rice variety was released in 2013 by the Bangladesh Rice Research Institute (BRRI dhan 62), which is claimed to contain 20–22 mg Zn kg^-1^ for brown rice. Nonetheless this is short of the target of 30 mg Zn kg^-1^ set by the HarvestPlus program (Shahzad et al., 2014). We suggest that Zn biofortification can be improved by drawing on under-utilized genetic materials, and by better understanding of Zn homeostasis of the rice plant.

## Conclusion

Zinc concentration in rice grains is influenced by plant-related factors (genetic factors) and environmental factors, and crop management strategies (agronomic factors). Greater understanding of how these factors interact to influence grain Zn accumulation is vital for enriching Zn concentration in rice grain. Improved Zn uptake and efficient remobilization are identified as key bottleneck for Zn biofortification. These bottlenecks should be addressed by exploiting the wide genetic diversity of rice germplasm. The rising atmospheric [CO_2_] is likely to reduce grain Zn concentrations and the underlying mechanism is not fully understood. Zn fertilization will also play an important role, especially where soils are inherently low in bioavailable Zn. Consequently, new genetic and management strategies need to be developed to minimize Zn deficiency for people whose staple diet is rice.

## Future Research Focus

Growing evidence suggests that wild and primitive rice has large and useful genetic variation in grain Zn concentration and that this variation is not yet fully exploited to improve grain Zn concentration and its bioavailability. Among them, Zn -efficient and -inefficient genotypes need to be evaluated under Zn sufficient and deficient conditions at different stages of growth and development to identify the genetic capacity for Zn uptake, utilization and loading into grain. Further, manipulation of Zn transporters and Zn ligands in the aleurone layer is likely to be a major target for biofortification of rice. The interaction of environmental and genetic factors on Zn homeostasis should also be established. Different processing technologies, and promoters and inhibitors of Zn bioavailability in rice grains, need special attention.

## Author Contributions

NN contributed 50% for the paper. All authors listed, have made substantial, direct and intellectual contribution to the work, and approved it for publication.

## Conflict of Interest Statement

The authors declare that the research was conducted in the absence of any commercial or financial relationships that could be construed as a potential conflict of interest.
